# Changes in Cystic Fibrosis Airway Microbial Community Associated with a Severe Decline in Lung Function

**DOI:** 10.1371/journal.pone.0124348

**Published:** 2015-04-21

**Authors:** Patrizia Paganin, Ersilia Vita Fiscarelli, Vanessa Tuccio, Manuela Chiancianesi, Giovanni Bacci, Patrizia Morelli, Daniela Dolce, Claudia Dalmastri, Alessandra De Alessandri, Vincenzina Lucidi, Giovanni Taccetti, Alessio Mengoni, Annamaria Bevivino

**Affiliations:** 1 Technical Unit for Sustainable Development and Innovation of Agro-Industrial System, ENEA (Italian National Agency for New Technologies, Energy and Sustainable Economic Development), Casaccia Research Center, Rome, Italy; 2 Cystic Fibrosis Microbiology and Cystic Fibrosis Center, Children's Hospital and Research Institute Bambino Gesù, Rome, Italy; 3 Department of Evolutionary Biology, University of Florence, Florence, Italy; 4 Department of Pediatrics, Cystic Fibrosis Center, G. Gaslini Institute, Genoa, Italy; 5 Department of Pediatrics, Cystic Fibrosis Center, Meyer Hospital, Florence, Italy; University of Tübingen, GERMANY

## Abstract

Cystic fibrosis (CF) is a genetic disease resulting in chronic polymicrobial infections of the airways and progressive decline in lung function. To gain insight into the underlying causes of severe lung diseases, we aimed at comparing the airway microbiota detected in sputum of CF patients with stable lung function (S) versus those with a substantial decline in lung function (SD). Microbiota composition was investigated by using culture-based and culture-independent methods, and by performing multivariate and statistical analyses. Culture-based methods identified some microbial species associated with a worse lung function, i.e. *Pseudomonas aeruginosa*, *Rothia mucilaginosa*, *Streptococcus pneumoniae* and *Candida albicans*, but only the presence of *S*. *pneumoniae* and *R*. *mucilaginosa* was found to be associated with increased severe decline in forced expiratory volume in 1 second (FEV_1_). Terminal-Restriction Fragment Length Polymorphism (T-RFLP) analysis revealed a higher bacterial diversity than that detected by culture-based methods. Molecular signatures with a statistically significant odds ratio for SD status were detected, and classified as *Pseudomonas*, *Burkholderia* and *Shewanella*, while for other Terminal Restriction Fragments (T-RFs) no species assignation was achieved. The analysis of T-RFLP data using ecological biodiversity indices showed reduced Evenness in SD patients compared to S ones, suggesting an impaired ecology of the bacterial community in SD patients. Statistically significant differences of the ecological biodiversity indices among the three sub-groups of FEV_1_ (normal/mild vs moderate vs severe) were also found, suggesting that the patients with moderate lung disease experienced changes in the airway assembly of taxa. Overall, changes in CF airway microbial community associated with a severe lung function decline were detected, allowing us to define some discriminatory species as well as some discriminatory T-RFs that represent good candidates for the development of predictive biomarkers of substantial decline in lung function.

## Introduction

Cystic fibrosis (CF) is the most frequent autosomal recessive life-threatening disease affecting 70,000 individuals worldwide, resulting in a progressive lung function decline. Currently, the percentage of predicted forced expiratory volume in 1 s (FEV_1_%) is commonly used to monitor lung function in CF and represents the best available predictor of survival for patients with CF [1], [2]. While a decline in lung function is common in almost all CF patients, the rate of decline is highly variable [3]. Several studies have evidenced that some CF patients’ FEV_1_ seriously decline despite antibiotic treatment [4] and that both long- and short-term fluctuations in lung function can be related to disease severity due to CFTR gene mutations, chronic bacterial infection, and periodic pulmonary exacerbation [3]. It has been found that, although the age and the FEV_1_% can help predict the relative severity of an individual CF phenotype, they do not necessarily provide a good prediction of the *risk* that a subject may run of a future rapid disease progression [5]. Interpreting the significance of changes in FEV_1_% over time first requires a more in-depth comprehension of the airway microbial community composition [6].

Recent evidence has revealed that CF airway infections are polymicrobial [7] and that the microbiota, as a collective entity, may contribute to pathophysiologic processes associated with chronic airway disease [8], [9]. It has been suggested that the bacterial community composition may be a better predictor of disease progression than the presence of stand-alone opportunistic pathogens [10]. Changes in airway bacterial community structures varied greatly upon exacerbations, decline in pulmonary health, antibiotic treatment, increase in patient age (for review, see [11], [12], [13]). According to Carmody and colleagues [14], certain genera appear to play an important role in driving change in the airway bacterial community composition at reacutization and, therefore, these might represent biomarkers for pulmonary exacerbation. Given the importance of lung function in CF patients health, it is by extension important to understand the complexity of CF microbiota in those patients showing a severe decline in lung function and identify those factors associated with higher/lower pulmonary function decline. To date, it is largely unknown which factors contribute to the loss in FEV_1_, especially in clinically stable CF patients. The presence of organisms not typically considered CF pathogens, in addition to the “typical” CF pathogens [15], may significantly affect the course and outcome of CF lung disease and may be responsible for the progressive decline in lung function [16]. Defining the microbial taxa associated with significant worsening of lung disease is only a first step in understanding their role in CF progression and provides novel insights into lung disease that could guide clinical management.

In this study we compared the airway microbiota detected in sputum from individual patients who have showed an important drop in FEV_1_% in the previous year (a rate of FEV_1_ decline greater than -5% predicted per year) and did not respond to conventional antimicrobial therapy (SD), versus that detected in sputum from stable (S) CF patients. All patients (S and SD) enrolled in the study were clinically stable, without any pulmonary exacerbation or antibiotic therapy (i.v. or oral) in the previous 4 weeks before specimen collection. The microbiota composition of a total of 78 patients attending three CF Centers in Italy was investigated by using culture-based methods, including anaerobic cultivation, Terminal Restriction Fragment Length Polymorphism (T-RFLP) analysis, and multivariate and statistical analysis. The primary objective was to achieve a better understanding of the species/taxon bacterial diversity in SD and S patients and the shifts in the dominant community members relative to lung function decline. Since in routine microbiology laboratories, microbial detection and identification traditionally rely on culture-dependent methods for both bacteria and fungi, we further aimed to evaluate the role of cultured yeast and filamentous fungi in a more rapid decline in pulmonary function and worsening of clinical outcomes.

## Materials and Methods

### Ethics Statement

Sputum samples from patients with CF were collected at Bambino Gesù Children's Hospital (Rome, Italy), Cystic Fibrosis Center, Meyer Children's Hospital (Florence, Italy) and Giannina Gaslini Children's Hospital (Genoa University, Genoa, Italy), in accordance with the ethical guidelines. The study was approved by the local Ethics Committees of each participating center [Prot. 85 of February 27, 2014 (Meyer Children’s University Hospital); Prot. n. 681 CM of November 2, 2012 (Bambino Gesù Children's Hospital); Prot. n FCC 2012 Partner 4-IGG of September 18, 2012 (Giannina Gaslini Institute)]. Informed written consent was obtained from all subjects aged 18 years and over and from parents of all subjects under 18 years of age prior to enrollment in the study. The study protocol was in accordance with the Guidelines of the European Convention of Human Rights and Biomedicine for Research in Children and to those of the Ethics Committees of Bambino Gesù, Meyer and Giannina Gaslini Hospitals. All measures were taken to ensure patient data protection and confidentiality.

### Patients

Seventy-eight patients were enrolled in the study between September 2012 and April 2013. These Institutions in Italy collectively provide care to a total of 680 patients (adult and children) with an average FEV_1_ decline of -1.44% predicted/year. Patients, who had been diagnosed with CF according to the published Guidelines [17], were treated according to current standards of care [18] with at least four microbiological controls per year [19]. Patients were eligible if they could be classified as clinically stable, without any pulmonary exacerbation or antibiotic therapy (i.v. or oral) in the previous 4 weeks before specimen collection [20], [21]. Since pulmonary function testing cannot generally be successfully performed until children reach 6 years of age, only CF patients older than 6 years were enrolled. The FEV_1_ measurements were performed more than 4 time/per year; in some cases, more than 20 measurements per year were performed, according to the clinical status of CF patients. Each FEV_1_ value was the average of at least three repeated FEV_1_ measurements obtained 2 to 3 min apart at a single testing session. The variability in FEV_1_% measures for each of 78 patients is reported in S1 Fig. The annualized rate of FEV_1_ decline was used to stratify patients. The rate of decline in pulmonary function was determined from each patient’s best percentage of predicted FEV_1_ (FEV_1_%) over the previous year. The difference between the best FEV_1_% registered in the previous year and the best FEV_1_% in the preceding year was considered to group patients. CF patients were categorized as “stable” (S), i.e. with a rate decline in FEV_1_ value not greater than -1,5% per year, and with a “substantial decline” (SD) in FEV_1_, i.e. a rate of FEV_1_ decline greater than -5% predicted per year, and not responding to the conventional antimicrobial therapy (chronic suppressive antibiotic and/or i.v. antibiotics treatments). In order to assess the influence of FEV_1_ status on the airway microbiota, S and SD patients were further categorized in three sub-groups: group I, CF patients with normal lung function or mild lung disease (FEV_1_% >70%); group II, CF patients with a moderate lung disease (70 ≥ FEV_1_% ≥ 40); group III, CF patients with a severe lung disease (FEV_1_% <40). FEV_1_ values were measured according to the American Thoracic Society—European Respiratory Society standards [22].

### Sample processing

The analysis of the bacterial community composition was performed on spontaneously expectorated sputum (SES) samples since sputum specimens represent by far the most widely used sample in productive patients [10]. Upon expectoration, CF sputum samples were immediately treated for 15 min with Sputolysin (Calbiochem, La Jolla, CA) in accordance with the manufacturer's instructions and split into aliquots for culture and molecular analyses. Aliquots for culturable analysis of anaerobic bacteria were transferred within 15 min to an anaerobic cabinet for processing, according to Tunney and colleagues [23]. Aliquots for culturable analysis of aerobic/microaerophilic bacteria and fungi were immediately examined, and the remaining aliquots were frozen and stored at -80°C for subsequent DNA extraction and molecular investigations.

### Bacteria and fungi detection by culture-methods

#### Media and growth conditions

To detect aerobic and facultative anaerobic microbes, 10 μl aliquots of 10-fold serial sputa dilutions up to 10^-6^ were prepared in 0.45% (w/v) NaCl and plated onto either Columbia agar with 5% sheep blood (CBA), MacConkey agar (MAC), Mannitol salt-agar (MSA), Chocolate agar with and without bacitracin (CHOC + BAC and CHOC), Columbia CNA agar with 5% sheep blood (CNA), Pseudosel agar (PA), *Burkholderia cepacia* Selective Agar (BCSA), or Brain heart infusion (BHI) agar. Plates were incubated at 37°C for 48 h aerobically, with the exception of CHOC, CHOC+BAC and CNA cultures, which were incubated in presence of 5% CO_2_ [24]. BCSA cultures showing no growth after 48 h of incubation were re-incubated for a further 5 days.

Anaerobic cultures were carried out by plating 10 μl aliquots of 10-fold serial sputa dilutions up to 10^-6^ prepared in quarter-strength Ringers lactate, supplemented with 0.05% (w/v) L-cysteine, on the following anaerobic media: CDC anaerobic blood-agar (CABA), Kanamycin-Vancomycin Laked Blood-Agar (KVLBA), Phenylethyl alcohol agar (PEA), Veillonella neomycin agar, Cadmium Sulfate Fluoride Acridine Trypticase (CFAT) agar or *Fusobacterium* selective agar (FSA). All plates were incubated anaerobically from 5 to 7 days at 37°C in an anaerobic work station (MACS MG-500, Don Whitley Scientific Ltd) with an atmosphere of 85% N_2_, 10% H_2_ and 5% CO_2_ at 37°C. Single colonies of each distinct morphotype were tested for oxygen sensitivity. Obligate anaerobes were defined as those isolates capable of growing anaerobically but not aerobically.

Yeasts and filamentous fungi recovery were performed by inoculating 10 μl aliquots of digested sample on Sabouraud Dextrose agar (SAB) with and without chloramphenicol (SAB + CAF and SAB). Plates were incubated for 14 days at 30°C.

#### Microorganism identification

All the colony morphotypes observed on the selective and non-selective media were identified by appearance (colonial morphology, pigment production, β-haemolysis on sheep’s blood agar, growth temperature), biochemical assays and/or proteomic profiling by matrix assisted laser desorption-time of flight mass spectrometry (MALDI-TOF MS) [25]. Both macroscopic and microscopic characteristics have been taken into account for the identification of yeasts and moulds. Ambiguous fungal strains were resolved by MALDI-TOF MS [26]. Aerobic and anaerobic bacterial isolates not resulting in species identification were characterized by means of molecular methods such as the amplification and sequencing of 16S rRNA and *recA* genes and species-specific PCRs [27], [28].

### DNA extraction

About 400 μl aliquots of frozen sputum were subjected to genomic DNA extraction using the Qiagen QIAamp DNA Mini Kit. Sample aliquots were spun at 10,000×g to pellet cellular material. After removal of the supernatant, cell pellets were re-suspended in 180 μl of the appropriate enzyme solution [20 mg/ml lysozyme (Sigma) in 20 mM Tris-HCl (pH 8.0), 2 mM EDTA and 1.2% Triton], incubated for 30 min at 37°C and then processed according to the manufacturer’s protocol. Quantity and purity of extracted DNA were checked by NanoDrop (NanoDrop Technologies, USA) and gel electrophoresis.

### PCR amplification, and T-RFLP profiling

The universal primers 926r (5’-CCG TCA ATT CAT TTG AGT TT-3’) and 8f-6FAM (5’-AGA GTT TGA TCC TGG CTC AG-3’) were used for amplification of the bacterial 16S rRNA gene [6] following a previously reported protocol [25]. Every sputum DNA sample was subjected to three independent PCRs, and the resulting products were pooled and purified by GE Healthcare Sephadex G-100 for T-RFLP analysis. Two hundred nanograms of each purified PCR product were digested with 10 units of *Cfo*I at 37°C for at least 5 h. Approximately 20 ng of digested PCR product were injected into an ABI 3730 DNA Analyzer (Applied Biosystems), using LIZ1200 (Applied Biosystems) as size standard.

Automated sequencing was performed by Genechron sequence service (Genechron Laboratory, Ylichron S.r.l., Rome, Italy).

### Statistical and bioinformatic analysis

T-RFLP profiles were processed with PeakStudio [26] to derive a matrix (Xt) with T-RFs sizes (binned at ±1 bp) and T-RFs intensities, as previously reported [27, 28], which allow an estimation of beta diversity indices. Culture-based identification were transformed in a binary matrix (Xc) for presence (1)/absence (0) of each taxon. Both T-RFLP and culture-based matrices were then used for subsequent statistical analyses. For community diversity parameters, Richness, Evenness and Shannon indices were computed, as implemented in the software Past [29] on Xt and Xc matrices.

Principal Component Analyses (PCAs) were computed on *Xt* and *Xc* matrices with the software R package [30] by using as centroids both taxa or TFRs in relation to the analysis of culturable microflora and T-RFLP, respectively. For biplot analyses, new matrices derived from *Xt* and *Xc* were produced by collapsing all samples from the same FEV_1_ group or pulmonary status (S and SD); 95% confidence ellipses were computed as scores for PCA. One-way ANOVA with Tukey *post-hoc* comparisons were performed with R package [30]. Conditional Maximum Likelihood Estimates (CMLE) of odds ratio (ORs) and 95% confidence Intervals (CI) were computed with OpenEpi suite (http://www.openepi.com). ORs were also estimated by applying a logistic regression model taking into account possible confounders, such as age, BMI, gender, CFTR genotype with R package.

Putative taxonomic assignment of T-RFLP peaks was then performed on the T-RFLP profiles by using the web platform MiCA [31] employing the T-RFLP Analysis (PAT+) option used to search for peak matching was performed on Ribosomal Database 10 (containing 1’519’357 bacterial 16S rRNA genes) with default parameters.

## Results

### Patients and FEV_1_ groups

A total of 78 CF patients (39 males and 39 females, mean age 26.99 years) were enrolled (40 S and 38 SD), according to their lung function (Table 1). Characteristics of S and SD cohorts, including CFTR genotype, gender, age, FEV_1_%, Body Mass Index (BMI), and nebulised antibiotics or oral azithromycin treatment are provided in S1 and S2 Tables.

**Table 1:** Demographic and clinical characteristics of all participants and in stable (S) and substantial-decliners (SD) status.

Characteristics	All Patients	Stables	Substantial-decliners
**Enrolled CF patients**	(n = 78)	(n = 40)	(n = 38)
**Sex (n)**	39 male	22 male	17 male
	39 female	18 female	21 female
***CFTR* genotype, n (%)**			
F508del/F508del	22 (28.20%)	11 (27.5%)	11 (28.95%)
F508del/other	34 (43.6%)	18 (45%)	16 (42.10%)
Other/other	22 (28.20%)	11 (27.5%)	11 (28.95%)
**Mean age ± SD**	26.99 ± 11.56	27.57 ±11.71	26.33 ±11.51
**Mean value of FEV**_**1**_ **% ± SD**	59.70 ± 25.02	64.35 ± 28.39	54.81 ± 20.13
**Disease stage categories, n (%)**			
Normal/mild (FEV_1_% > 70)	30 (38.46%)	17 (42.5%)	13 (34.21%)
Moderate (70 ≥ FEV_1_% ≥ 40)	27 (34.62%)	14 (35%)	13 (34.21%)
Severe (FEV_1_% < 40)	21 (26.92%)	9 (22.5%)	12 (31.58%)

### Culture microbiota in S and SD patients

Seventy-eight specimens, obtained during the course of routine medical care, in accordance with the ethical Guidelines, were processed by culture-dependent approaches, including the classical microbiological approach in accordance with the Guidelines for CF sputum analysis and advanced approaches for the identification of bacterial isolates, anaerobic bacteria, and fungi. The occurrence of all microbial taxa, not only those known to be involved in pulmonary infections, was used to determine if a single taxon or an assemblage of them may be associated with SD or S status.

**Fig 1:**
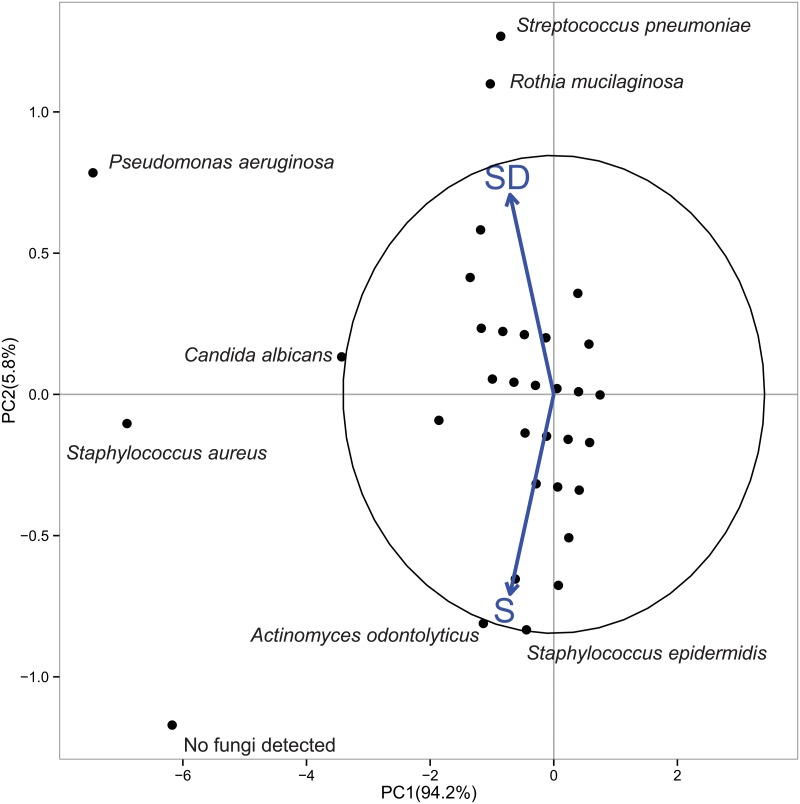
Biplot of the principal component analysis of culturable taxa from S and SD patients. Ellipse with 95% is reported. The numbers on the axes indicate the amount of variance explained by each component. The labels outside the 95% ellipse have been jittered to avoid overlaps. Black circles represent culturable taxa. Are indicated those taxa, ouside the ellipse, that are significantly associated to the PC1 and PC2.

Principal Component analysis (PCA) of the culturable microflora detected from S and SD samples did not reveal any clear distinction between S and SD groups, neither among the three different clinical conditions examined (I, normal/mild lung disease; II, moderate disease; III, severe disease) (data not shown). Interestingly, when PCA was performed using the detected taxa as centroids, differences in the microbial community composition between S and SD groups were found. Fig 1 shows that *Pseudomonas aeruginosa*, *Rothia mucilaginosa*, *Streptococcus pneumoniae*, and *Candida albicans* for SD group, the absence of fungi and *Staphlyoccoccus aureus* for S group were the most important taxa contributing to variance differences among S and SD patients in our dataset (in terms of taxa outside the confidence ellipse 95%, which groups the most similarly occurring taxa).

To statistically evaluate the relationships between the differential presence of these taxa and the patients’ status, ORs were computed (Tables 2 and 3). Statistically significant ORs for SD were found for the presence of *S*. *pneumoniae* (OR = 9.954) and *R*. *mucilaginosa* (OR = 4.867) (Table 2). However, when the possible confounders were taken into account in a logistic regression (S3 Table) for *S*. *pneumonia*, OR was 1.12 (CI 0.14–6.11) and *R*. *mucilaginosa* OR was 7.37 (CI 1.45–44.41). In the same model *P*. *aeruginosa* OR was 1.14 (CI 0.34–3.87), while *C*. *albicans*, *S*. *aureus* and absence of fungi had ORs of 2.30, 1.04 and 0.57, respectively.

**Table 2:** Odds ratio from culturable microflora with differential occurrence in SD and S patients.

Taxon	OR	CI 95%	
		lower	upper
*Streptococcus pneumonia*	**9.95**	**1.47**	**233.9**
*Rothia mucilaginosa*	**4.87**	**1.03**	**38.8**
*Pseudomonas aeruginosa*	1.66	0.657	4.202
*Candida albicans*	1.04	0.388	2.79
*Staphylococcus aureus*	0.86	0.34	2.18
absence of fungi	0.77	0.30	1.96

Data report the taxa detected from Principal Component Analysis (Fig 1), the odds ratio (OR) of association between presence of the taxa and SD status, the 95% confidence intervals (CI 95%). Statistically significant ORs are reported in bold.

**Table 3:** Odds ratio from culturable microflora with differential occurrence in FEV_1_ groups.

Taxon	OR	CI 95%	
		lower	upper
*Candida albicans*	1.57	0.526	4.549
*Pseudomonas aeruginosa*	1.46	0.397	4.942
No anaerobic bacteria	1.46	0.398	4.942

Data report the taxa detected from Principal Component Analysis (Fig 1), the odds ratio (OR) of association between FEV_1_ values in the FEV_1_ group III with respect to a cohort composed by FEV_1_ group I and FEV_1_ group II patients, the 95% confidence intervals (CI 95%). Statistically significant ORs are reported in bold. FEV_1_, group I = normal/mild (FEV_1_%>70); FEV_1_, group II = moderate (70≥FEV_1_%≥40); FEV_1_, group III = severe (FEV_1_%<40).

**Fig 2:**
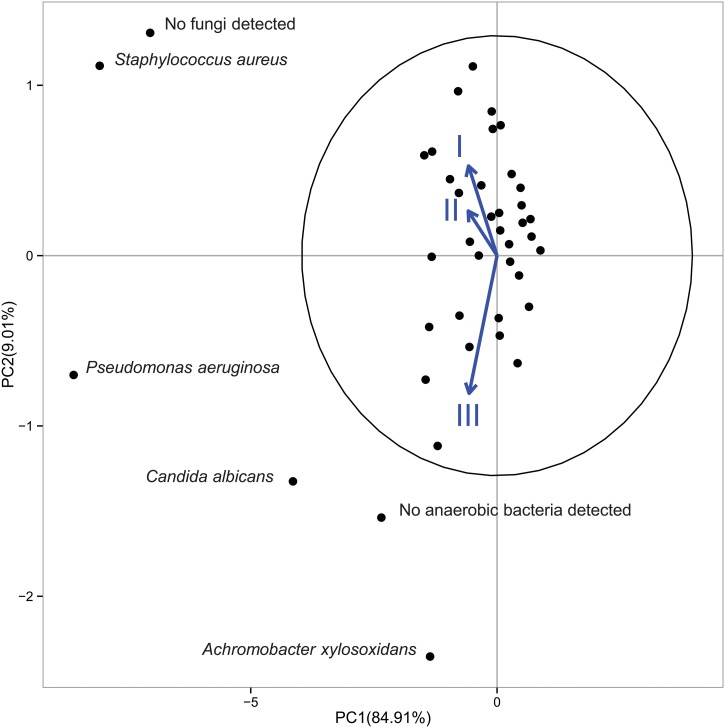
Biplot of the principal component analysis of culturable taxa from 78 specimens of patients with CF considering their FEV_1_ groups. Ellipse with 95% is reported. The numbers on the axes indicate the amount of variance explained by each component. The labels outside the 95% ellipse have been jittered to avoid overlaps. Black circles represent culturable taxa. Are indicated those taxa, ouside the ellipse, that are significantly associated to the PC1 and PC2.

When the three different FEV_1_ groups were considered, PCA showed a differential occurrence of *Achromobacter xylosoxidans*, *P*. *aeruginosa*, *C*. *albicans* as well as the absence of an anaerobic microflora in CF patients with severe lung disease (group III) (Fig 2). Conversely, no statistical support could be obtained for the presence of *A*. *xylosoxidans*, since this species was detected in only one patient of group III. When ORs for the FEV_1_ of group III were considered, in comparison with FEV_1_ I and II groups (Table 3), no statistically significant ORs were detected between culturable microflora and FEV_1_ decline. By applying a logistic regression including confounders the same result was found (S4 Table), with no detectable limit for the lower confidence interval.

There were no statistically significant differences in terms of overall values of diversity indices, in alpha diversity values found between the whole groups of SD and S patients (Table 4) nor between SD and S patients belonging to the severe group (FEV_1_ group III), nor among the three FEV_1_ sub-groups in both SD and S patients (data not shown). Furthermore, no significant correlation was found between diversity indices and FEV_1_ values (as Pearson’s r, p value was > 0.05 for all indices).

**Table 4:** Diversity estimates for cultured bacterial communities in sputum samples of CF patients.

CF patients	Diversity indices		
	Shannon	Evenness	Richness
SD	1.64±0.39	0.37±0.06	5.6 ±2.3
S	1.65±0.40	0.36±0.06	5.7 ±2.4

Data show the mean indices for substantial decliners (SD) and stable (S) patients ± standard deviation. Data were analysed by one-way ANOVA and Tukey *post-hoc* comparison. No statistically significant differences were found.

### Total microbiota in S and SD CF patients

#### Community composition: species absence/presence

In the 78 sputum samples analyzed by T-RFLP, a total of 1411 bands representing 208 different T-RF lengths were detected. The number of individual bands in each sample ranged from 2 to 43. In particular, ranges were from 3 to 43 for the S patients and 2 to 39 for the SD patients. The mean number of T-RF bands per patient in S sample set (20.2+/-11.4) was higher than that of the SD sample (15.5+/-10.2), though the difference was not significant (P<0.06). Among the 208 T-RF bands detected, 82 (39.4%) were “singletons,” defined here as bands that occurred in only one sample. Singletons were detected in 18 (45%) S and 10 (27.2%) SD samples.

#### Bacterial community structure

**Fig 3:**
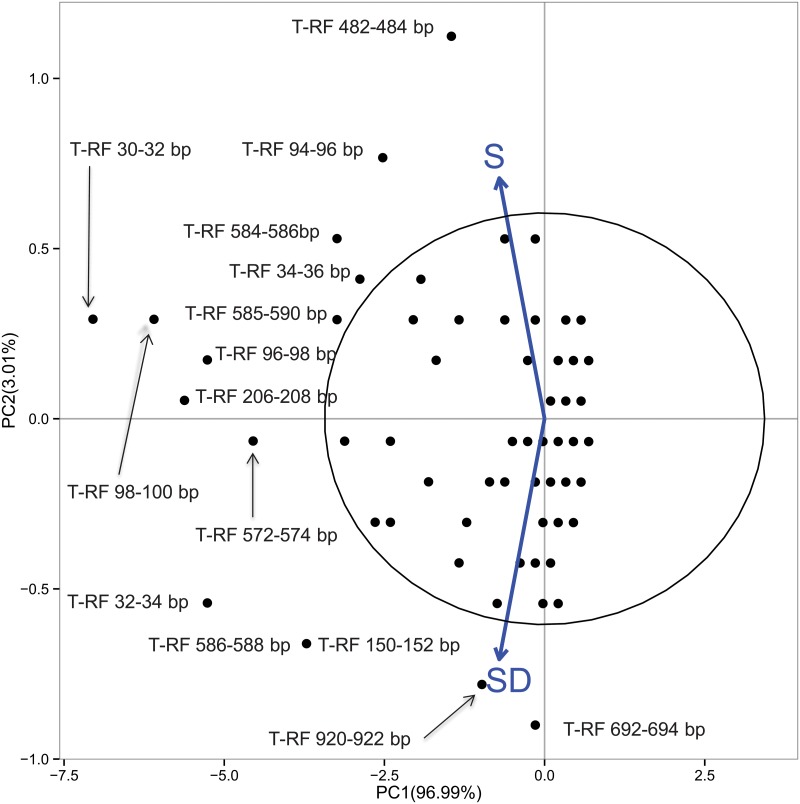
PCA of total occurrences of T-RFs in CF patients showing comparison of S with SD patients. Ellipse with 95% is reported. The numbers on the axes indicate the amount of variance explained by each component. The labels outside the 95% ellipse have been jittered to avoid overlaps. Black circles represent T-RFs. Are indicated those T-RFs, ouside the ellipse, that are significantly associated to the PC1 and PC2.

PCA carried out on single patient’s T-RFLP profiles did not reveal any clear differences between S and SD patients, nor any differences associated with lung disease status (S2 Fig). When PCA was performed on the markers (T-RFs) as centroids, several (16) T-RFs with a differential contribution toward SD and S (as those outside the confidence ellipse at 95%) were found (Fig 3).

One T-RF (920–922 nt) resulted in a higher impact on the variance of SD status, while another T-RF (482–484 nt) contributed more to the variance of S status. Moreover, the T-RF at 692–694 nt was only detected in SD patients (in eight out of 38 patients, corresponding to approx. 21%). To statistically evaluate the relationships between the differential presence of these molecular markers (T-RFs) and the patients’ status, ORs were computed for TFs, too (Table 5). In particular, some T-RFs with OR>1 (i.e., more present in SD than in S patients) were detected. Here a statistically significant OR was found for T-RF 920–922 nt (putatively attributed to *Shewanella*, and *Burkholderia*).The logistic regression model was well in agreement with CMLE ORs estimates (S5 Table).

**Table 5:** Odds ratio from T-RFs with differential occurrence in S and SD patients.

TRF size (nt)	OR	CI 95%	
SD *vs* S patients		lower	upper
30–32	0.94	0.26	3.39
32–34	2.05	0.79	5.51
34–36	0.79	0.31	1.98
94–96	0.56	0.21	1.43
96–98	0.28	0.06	1.06
98–100	0.93	0.33	2.64
150–152	2.04	0.83	5.15
206–208	1.18	0.44	3.17
482–484	**0.29**	**0.08**	**0.88**
572–574	1.25	0.50	3.13
574–576	1.04	0.40	2.68
584–586	0.72	0.29	1.79
586–588	2.04	0.83	5.15
588–590	0.89	0.36	2.20
692–694	n.d.	n.d.	n.d.
920–922	**3.61**	**1.06**	**14.37**

Data report the T-RFs size in nucleotides (nt), the CMLE odds Ratio (OR) estimates of association between presence of the T-RF and SD status, and the 95% confidence intervals (CI 95%). The reported attributions indicate the main hits retrieved for that particular size. Statistically significant ORs are reported in bold.

**Fig 4:**
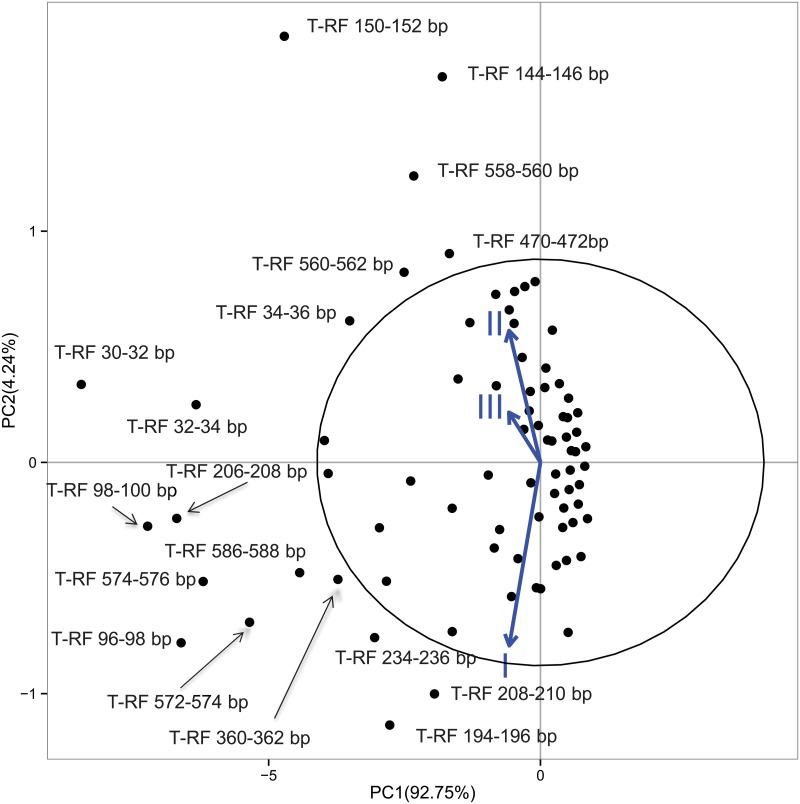
PCA of total occurrences of T-RFs in CF patients showing comparison among the FEV_1_ groups. Ellipse with 95% is reported. The numbers on the axes indicate the amount of variance explained by each component. The labels outside the 95% ellipse have been jittered to avoid overlaps. Black circles represent T-RFs. Are indicated those T-RFs, ouside the ellipse, that are significantly associated to the PC1 and PC2.

PCA biplot (Fig 4) showed that the vector of FEV_1_ group I has a different orientation with respect to those of the other FEV_1_ groups, suggesting that some T-RFs can indeed be differentially present. We then computed ORs for the 18 T-RFs outside the ellipse 95%. Results are reported in Table 6. One T-RF (96–98 nt) was shown to contribute more to the variance of FEV_1_ group III, while three T-RFs (558–560 nt, 144–146 nt, 150–152 nt) contributed more to the variance of FEV_1_ I and II groups. However, all these T-RFs but 558–560 nt (putatively attributed to *Gammaproteobacteria*, with matching on *Halomonas*) could not be assigned to bacteria taxonomy by MiCA (http://mica.ibest.uidaho.edu). However, though CMLE ORs (Table 6) showed significant ORs, the logistic regression model (S6 Table) did not support the data.

**Table 6:** Odds ratio from T-RFs with differential occurrence in FEV_1_ groups.

TRF size (nt)	OR	CI 95%	
FEV_1_ (III) *vs* FEV_1_ (I) + (II) patients		Lower	upper
208–210	2.36	0.84	6.74
194–196	2.55	0.96	6.87
234–236	2.09	0.80	5.52
572–574	2.11	0.81	5.77
96–98	**3.00**	**1.00**	**10.18**
574–576	2.15	0.78	6.34
586–588	1.50	0.59	3.86
360–362	1.47	0.57	3.77
98–100	2.10	0.68	7.23
206–208	1.66	0.59	4.94
32–34	1.01	0.38	2.73
30–32	1.22	0.33	5.06
34–36	0.70	0.26	1.83
560–562	0.36	0.11	1.09
470–472	0.40	0.10	1.33
558–560	**0.28**	**0.07**	**0.90**
144–146	**0.07**	**0.00**	**0.42**
150–152	**0.25**	**0.09**	**0.66**

Data report the T-RFs size in nucleotides (nt), the CMLE odds Ratio (OR) estimates of association between presence of the T-RF in FEV_1_ group III *vs* a cohort composed by FEV_1_ group I + FEV_1_ group II patients, and the 95% confidence intervals (CI 95%). The reported attributions indicate the main hits retrieved for that particular size. Statistically significant ORs are reported in bold.

#### Diversity indices

To investigate changes in bacterial communities that may have occurred with severe decline in lung function, diversity indices were computed for all samples by using community ecology parameters (beta diversity estimates). Results revealed that the Evenness, i.e. the equal distribution of T-RFs, resulted higher in S sample set (0.52±0.18) than in SD sample set (0.43±0.18) (p<0.05). The other diversity indices considered (Richness and Shannon) were not statistically different between the whole dataset of S and SD patients (p>0.05) (Table 7a), and no statistically significant changes were observed between S and SD groups when considering the three FEV_1_ sub-groups separately (data not shown). The diversity indices (Evenness, Richness) values did not allow detection of differences among the three FEV_1_ groups (Table 7b), while significant (p<0.05) differences in Shannon indices of FEV_1_ (II) *vs* FEV_1_ (I) and of FEV_1_ (II) *vs* FEV_1_ (III) were found. However, no significant differences were found for FEV_1_ (I) *vs* FEV_1_ (III).

**Table 7:** Diversity estimates for total bacterial communities in sputum samples of CF patients.

Bacterial communities	Diversity indices		
**A) T-RFLP community profiles**	**Shannon**	**Evenness**	**Richness**
SD	1.89 ±0.55	0.43 ±0.18^a^	19.8 ±11.0
S	1.86 ±0.56	0.52 ±0.18^b^	16.5 ±10.8
**B) T-RFLP profiles / FEV**_**1**_ **groups**			
FEV_1_ group I	1.95±0.49^a^	0.45±0.17	19.7±9.5
FEV_1_ group II	1.59±0.48^b^	0.47±0.22	15.1±11.9
FEV_1_ group III	2.09±0.61^a^	0.53±0.15	19.6 ±11.6

Data show the mean indices for substantial decliners (SD) and stable (S) patients ± standard deviation. Different superscript letters in a column indicate statistically significant (P<0.05) differences after one-way ANOVA and Tukey *post-hoc* comparison.

## Discussion

In the present study, we used both culture-dependent methods for detection of aerobic and anaerobic bacteria and fungi, and a culture-independent method by terminal restriction fragment length polymorphism (T-RFLP), which provide a fingerprint of the most highly abundant members of the community. Data presented here provide new insights into the CF airway microbiota of S and SD patients, population dynamics of the microbiota following lung disease status (normal/mild, moderate and severe lung disease), the ecology of the bacterial communities related to lung function decline and the establishment of specialized communities of pathogens associated with poor pulmonary function, including putative discriminant microbial species and T-RFs for S and SD groups.

The rate at which FEV_1_% declines over a period is an indicator of aggressiveness of a patient’s lung disease, regardless of the stage of the patient’s lung disease (normal/mild, moderate and severe) [32]. In the present work, we defined a decrease of 5% or more in FEV_1_ predicted per year as a substantial decline in lung function. This percentage drop in FEV_1_ suggests a substantial decline similar to that defined by Vandenbranden and colleagues [33] and twice the one reported in published studies [4]. As pointed out by Cazzola and colleagues [34]), a change of 5% or more from baseline values of FEV_1_ is considered to be clinically important also in chronic obstructive pulmonary disease. Therefore, the microbiota investigation in CF patients with a more substantial decline in lung function over the previous year can be very useful in order to try to understand where a pathogen is just a marker of disease severity or an independent contributor to the loss of lung function [6]. As the ultimate goal of pulmonary interventions is to retard disease progression and therefore reduce the rate of lung function decline, defining new potential biomarkers for the early detection of severe rate of decline in lung function could be essential in making rational decisions regarding intervention.

The presence of *P*. *aeruginosa* in respiratory tract cultures has been previously reported to be associated with a more rapid decline in lung function, especially when mucoid phenotype is present [35]. However, Vandenbranden and colleagues [33] found that the presence or absence of *P*. *aeruginosa* or mucoid *P*. *aeruginosa* was not predictive of lung function decline. Our findings from culture-based analysis showed that *P*. *aeruginosa* cannot be strongly proposed as indicative of severe decline in lung function (OR = 1.14, CI 0.34–3.87). It is noteworthy that maintenance treatment for chronic *P*. *aeruginosa* with inhaled antibiotics and/or azithromicin in accordance with the Guidelines [36] resulted in slowing down the rate of decline in FEV_1._ Our culture-based results suggest an important role of *S*. *pneumoniae* and *R*. *mucilaginosa* as marker species for SD status. Although *S*. *pneumoniae* is a common respiratory tract pathogen, it is unusual in CF [37]. This bacterial species is able to persist in the CF lung and has been recently regarded as pathogenic within the CF community [38]. However, to the best of our knowledge, it is still unclear whether *S*. *pneumoniae* has a role in acute exacerbation and severe lung decline or can act synergically with other CF pathogens. *R*. *mucilaginosa* was previously reported as an aerobic species isolated from CF sputum [23] and was defined by several authors [39] [40] as a “new” emerging CF pathogen. Recently, Lim and colleagues [41] confirmed that *R*. *mucilaginosa* is present and metabolically active in the lungs of CF patients and that it evolves and adapts to each patient’s lung environment over the course of a persistent infection. Our results also reinforce the previous findings of Chotirmall and colleagues [42] who reported that colonization with *C*. *albicans* presages FEV_1_ decline in CF. Although the same authors suggested the implication of *C*. *albicans* in the decline of CF lung function, the clinical relevance of yeasts is still a matter of debate, and has yet to be confirmed. Our findings add support to (i) the pathogenicity of species derived from the oral cavity and usually considered as clinically insignificant, and (ii) the complex interaction between typical pathogens and microbiota, such as the association between *P*. *aeruginosa* and anaerobes/fungi.

It is well known that CF airways harbor numerous organisms that evade detection by culture-based methods [43]. To carefully examine the microbiota from patients with stable versus worsening disease status, we chose to utilize T-RFLP analysis to generate community profiles. T-RFLP is a popular molecular profiling technique with the capacity to resolve community members based on the position of restriction sites in the 16S rRNA gene [44]. T-RFLP profiles have been extensively used for community differentiation, identification of specific organisms in microbial populations and comparison of the relative phylotype richness and community structure in a diverse range of environments [45], [46], [47], [48], [49]. When previously applied to the analysis of spontaneously expectorated sputum from CF patients, T-RFLP has indicated the presence of a diverse array of bacterial species [7], [10], [50], [51]. In this study, we used T-RFLP analysis as a tool to investigate two key topics. First, do the bacterial communities (microbiota) detected in S samples differ from those in SD samples? Additionally, are there differences in the microbiota among CF patients with different status of lung disease? Results obtained in the present study by T-RFLP analysis revealed a greater bacterial diversity within sputum samples than that detected by culture approaches. We did not find T-RFLP profiles exclusive of the patients from S or SD groups. T-RFLP analysis revealed sixteen T-RFs as the most differentiating between S and SD groups after PCA which could be potentially exploited as novel molecular DNA-based markers for S *vs* SD discrimination. However, in our dataset, significant ORs were only recorded for two of them (and only one was possible to identifiy as putative *Shewanella*). Interestingly, the other T-RFs which differentiate S from SD (though not significantly on the basis of ORs) were putatively identified known CF pathogens, *Pseudomonas* (206–208 nt; 572–574 nt) and *Burkholderia* (30–32 nt; 32–34 nt), but most of them resulted in no species assignation (only ~18% of T-RF band lengths match a band length generated from published sequence data). Therefore, these unknown T-RFs could be either novel species or related species/strains of the core community of CF airways, but with enough heterogeneity in the 16S rRNA gene that may generate slightly different fragment sizes [52]. *S*. *pneumonia* and *R*. *mucilaginosa* were detected by culturing but not by T-RFLP analysis. It is well known that molecular methods based on 16S sequence data have a limited ability to resolve taxonomic identification at the species level [53] and may introduce biases during the primer-binding step of PCR [54]. In spite of these limits, and taking also into consideration that T-RFLP only allows a putative (though fast) taxonomic assignment, this culture-independent method adds valuable information to the data obtained from culture analysis. To gain a more comprehensive characterization of CF lung microbial communities, more sophisticated and expensive techniques, such as bacterial metabarcoding [55] by 16S rDNA and metagenomic sequencing [56], [57] will be applied.

To investigate changes in bacterial communities that may have occurred with a substantial decline in lung function, the diversity indices on T-RFLP data were calculated for all samples. Diversity provides a measure of community complexity based on the number (Richness) and relative abundances (Evenness) of the species present [44]. The reduced Evenness observed in SD respect to S group of patients revealed an impaired ecology of the bacterial community in SD patients, which in turn can be associated with lung function decline experienced by those patients. When we assessed community structures to describe the degree of change occurring in the airway microbiota following the decline in lung function, we did not find a decrease in Shannon diversity index with advanced disease in the SD group. As suggested by Zhao and colleagues [32], other factors, such as antibiotic use, rather than lung function or patient age have been found to be the primary drivers of the decreasing bacterial diversity in CF patients with progressive lung disease. In our study, patients with a more rapid decline in lung function did not have a higher antibiotic exposure over the previous year with respect to S patients; additionally, neither S nor SD patients received antibiotics in the thirty days preceding the specimen collection. Collectively, our results suggest that the antibiotic use is not likely to have contributed to the failure in detecting a decrease in community diversity with advanced disease in the SD group. Moreover, we cannot *a priori* exclude that the use of relative peak intensities as a proxy for taxa frequency, though largely used (for instances see [52], [58], [59]), this may be someway biased and can produce altered data. Our analyses suggest that overall bacterial diversity remains relatively stable despite the decreasing in relative abundances of the species in SD patients. Indeed, as previously stated by Zhao and colleagues [32], community diversity alone is not a sufficient indicator of the disease status. However, considering only the three subgroups of FEV_1_ (normal/mild *vs* moderate, *vs* severe), independently from the rate at which FEV_1_% declines, statistically significant differences among the three sub-groups were also found, suggesting that the patients with intermediate FEV_1_ values are experiencing changes in the airway assembly of taxa. A more detailed taxonomic and functional analysis of the microbial community of lung microbiota of CF patients could help elucidating the microbial factors that can contribute to such changes.

In conclusion, by combining culture-dependent and culture-independent methods with ecological tools and clinically relevant information, a more comprehensive view of microbial community composition in SD patients with CF was determined. Overall, our results suggest that the presence of *P*. *aeruginosa* “per se”, as well as the single T-RFLP profiles, are not able to define the S or SD group. New biomarkers are required in CF to predict the decline clinical phenotype and to monitor the response of CF patients to existing and new therapeutic strategies. Microbial factors represent a potential to provide biomarkers for the early detection of the substantial lung function decline in CF. We have identified some discriminatory species as well as some discriminatory T-RFs, that represent good candidates as predictors of substantial decline in lung function, enabling the stratification of individuals with CF at high or low risk of future severe lung function decline. However, only longitudinal studies can help determine patterns of association between the CF airway microbiome and lung disease. A more in-depth investigation of microbial airway bacterial communities in longitudinal studies and by high-throughput sequencing and bioinformatics tools will provide powerful means to better understand the contribution of the airway microbiome to severe decline in FEV_1_ and its potential for the development of new biomarkers as predictors of severe pulmonary disease in CF patients.

## Supporting Information

S1 Fig Box plot showing the different distributions of FEV_1_ expressed as a percentage of the predicted value (FEV_1_%) in each of 78 patients (S and SD). The top and bottom boundaries of each box indicate 75th and 25th quartile values, respectively, and black lines inside each box represent 50th quartile (median) values. Ends of the whiskers mark the lowest and highest FEV1 value of the FEV_1_ measurements. For both S and SD patients is possible to follow the decreased FEV_1_ values passing from sub-group I (normal/mild, FEV_1_%>70) to sub-group III (severe lung disease, FEV_1_%<40).
(PDF)

S2 Fig Principal Component Analysis of T-RFLP profiles of 78 patients with CF. Blue dots, stable patients; red dots, substantial-decliners patients. The numbers on the axes indicate the amount of variance explained by each component.
(PDF)

S1 Table Characteristics of the stable cohort at the enrollment in the study. (PDF)

S2 Table Characteristics of the substantial decliners’ cohort at the enrollment in the study. (PDF)

S3 Table Odds ratio estimates of SD vs. S for cultured microflora. (XLSX)

S4 Table Odds ratio estimates of FEV1 III vs. FEV1 I+II for cultured microflora. (XLSX)

S5 Table Odds ratio estimates of SD vs. S for T-RFLP profiles (total microflora). (XLS)

S6 Table Odds ratio estimates of FEV1 III vs. FEV1 I+II for T-RFLP profiles (total microflora). (XLS)
