# Supplementary figures and images for: Changes in Cystic Fibrosis Airway Microbial Community Associated with a Severe Decline in Lung Function
Source: PLoS One. 2015 Apr 21;10(4):e0124348. doi: 10.1371/journal.pone.0124348 (PMC4405530; doi:10.1371/journal.pone.0124348)

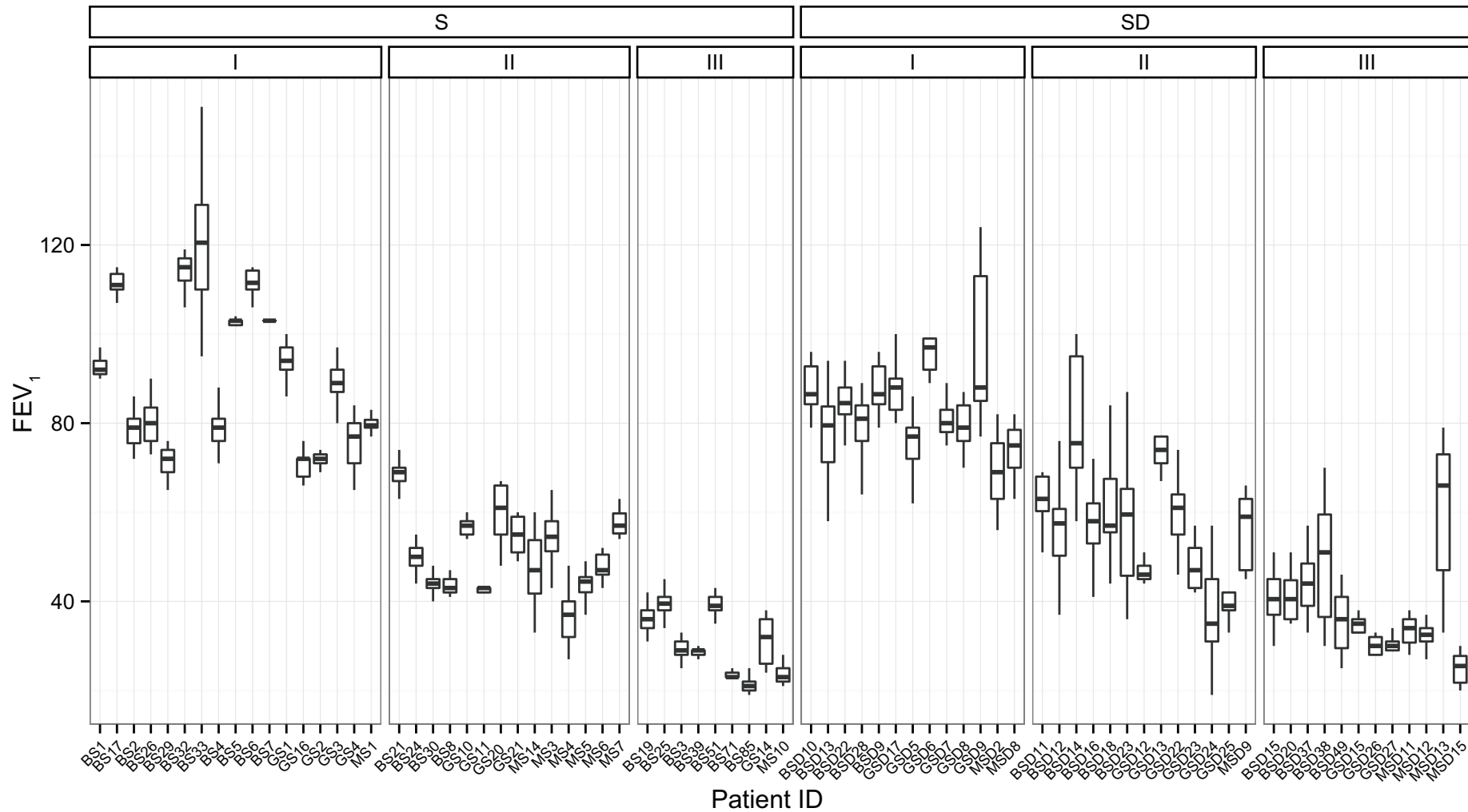

Supplement: S1 Fig — The top and bottom boundaries of each box indicate 75th and 25th quartile values, respectively, and black lines inside each box represent 50th quartile (median) values. Ends of the whiskers mark the lowest and highest FEV1 value of the FEV1 measurements. For both S and SD patients is possible to follow the decreased FEV1 values passing from sub-group I (normal/mild, FEV1%>70) to sub-group III (severe lung disease, FEV1%<40). (PDF) [file pone.0124348.s001.pdf]

Component 2 (14.5%)

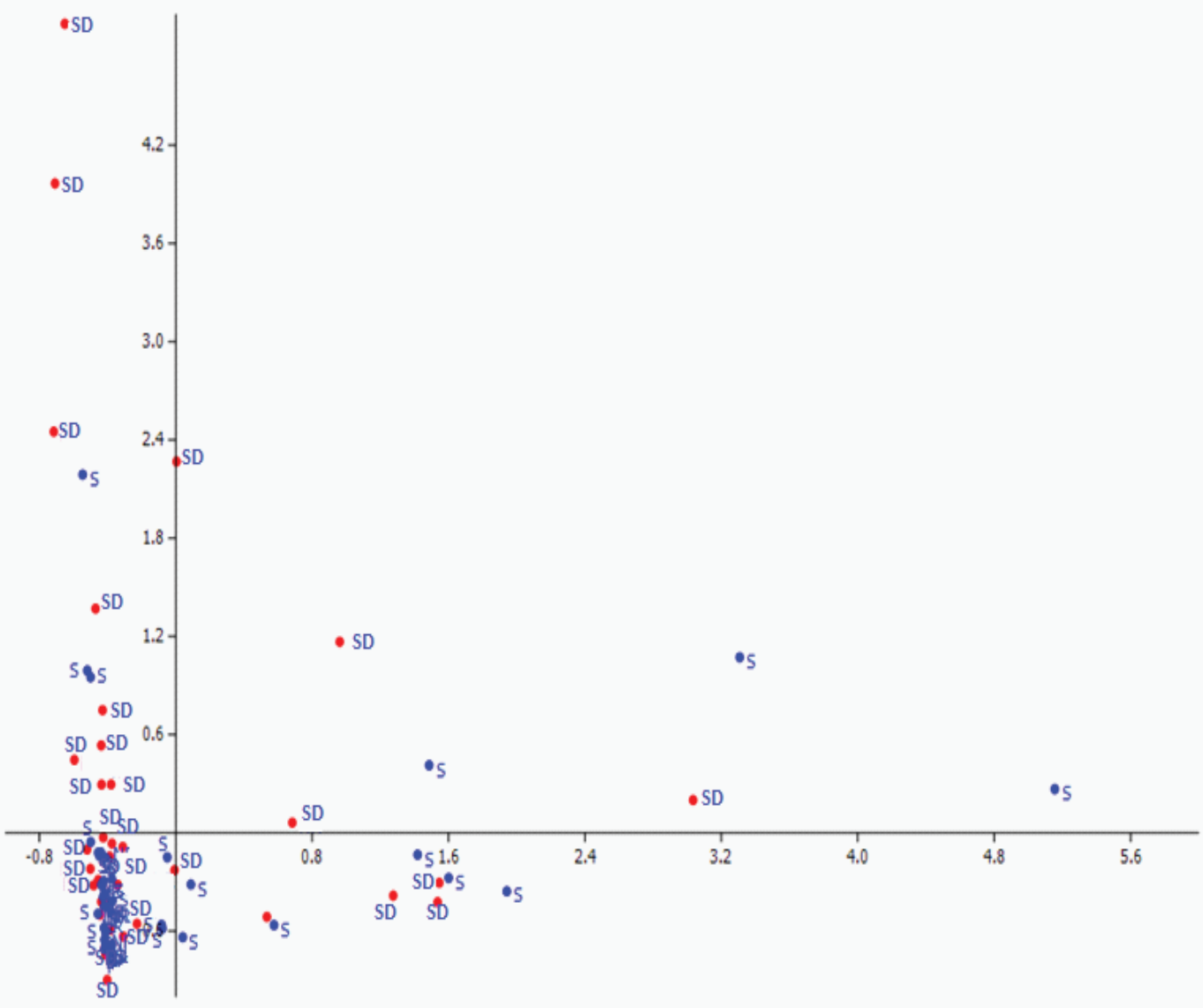

Supplement: S2 Fig — Blue dots, stable patients; red dots, substantial-decliners patients. The numbers on the axes indicate the amount of variance explained by each component. (PDF) [file pone.0124348.s002.pdf]
