# Supplementary material for: Changes in Cystic Fibrosis Airway Microbial Community Associated with a Severe Decline in Lung Function
Source: PLoS One. 2015 Apr 21;10(4):e0124348. doi: 10.1371/journal.pone.0124348 (PMC4405530; doi:10.1371/journal.pone.0124348)
Supplement: S1 Table — (PDF) [file pone.0124348.s003.pdf]

Table S1. Characteristics of the stable cohort at the enrollment in the study.

Study ID[a]	Age (years)	Gender	CFTR Genotype	FEV$_1$ (%predicted)	BMI	Microflora	Maintenance antimicrobial therapy
BS1 (I)	32	M	F508del/N1303K	94	23.8	*Pseudomonas aeruginosa, Aspergillus* spp.	aerosolized aztreonam aerosolized colistimethate,
BS2 (I)	11	F	F508del/F508del	78	17.0	*P. aeruginosa*	aerosolized tobramycin
BS4 (I)	29	F	G542X/S549R(A›C)	79	21.0	*Staphylococcus aureus*, *Serratia marcescens, Scedosporium prolificans*	aerosolized colistimethate, azithromycin
BS5 (I)	12	M	F508del/G91G	103	19.5	*S. aureus, Candida albicans*	none
BS6 (I)	17	F	F508del/R117H	112	22.0	*P. aeruginosa, Prevotella melaninogenica, Veillonella parvula, Neisseria perflava, Neisseria flavescens, Haemophilus parainfluenzae, Streptococcus salivarius*	aerosolized tobramycin
BS7 (I)	12	M	F508del/G91G	103	19.5	*S. aureus, P. melaninogenica, Haemophilus influenzae, N. perflava, Streptococcus* spp.	none
BS17 (I)	28	F	F508del/W1282X	112	25.0	*P. aeruginosa*, *Staphylococcus epidermidis, Staphylococcus haemolyticus, Granulicatella adiacens, C. albicans*	aerosolized colistimethate
BS26 (I)	20	F	F508del/2789+5G›A	80	21.1	*Stenotrophomonas maltophilia*, *Corynebacterium durum Actinomyces odontolyticus, Capnocytophaga sputigena, H. parainfluenzae, S. parasanguinis, C. albicans*	aerosolized colistimethate
BS29 (I)	24	F	F508del/L1077P	72	23.1	*S. maltophilia*, *S. aureus, A. odontolyticus*	aerosolized tobramycin
BS32 (I)	11	M	N1303K/G1244E	115	16.0	*S. maltophilia, S. aureus, V. V. parvula, Veillonella dispar, Veillonella atypica, Prevotella denticola, P. melaninogenica, Lactobacillus* spp., *S. salivarius, S. parasanguinis*	aerosolized tobramycin, azithromycin
BS33 (I)	8	F	N1303K/G1244E	120	21.9	*S. aureus, V. dispar, C.*	aerosolized tobramycin

Study ID	Age	Gender	CFTR Genotype	FEV_1	BMI	Microflora	Maintenance antimicrobial therapy
						sputigena, Rothia aeria, Streptococcus pneumoniae, Streptococcus sanguinis, N. perflava	
GS1 (I)	13	M	R1162X/UK	100	20.1	*S. aureus, Propionibacterium acnes, Gemella haemolysans, C. albicans*	aerosolized colistimethate, azithromycin
GS2 (I)	44	F	F508del/R334W	72	20.7	*P. aeruginosa, S. aureus, A. odontolyticus, V. atypica, G. haemolysans, Streptococcus oralis*	none
GS3 (I)	30	F	F508del/F508del	87	21.0	*S. aureus, Achromobacter xylosoxydans, P. denticola, S. oralis, Streptococcus pseudopneumoniae, Streptococcus peroris*	none
GS4 (I)	40	F	F508del/F508del	84	19.2	*P. aeruginosa, S. aureus,* Enterobacteriaceae, *V. atypica, C. albicans*	aerosolized colistimethate, azithromycin
GS16 (I)	38	M	F508del/F508del	81	22.2	*S. aureus, Prevotella nanceiensis, A. odontolyticus, C. albicans, Aspergillus fumigatus*	aerosolized tobramycin
MS1 (I)	30	F	G1244 E/G1244 E	83	22.9	*P. aeruginosa, S. aureus, P. acnes, S. oralis, S. pseudopneumoniae, S. peroris*	aerosolized colistimethate
BS8 (II)	19	M	W1282X/621+IG›T	43	15.3	*A. xylosoxidans, Fusobacterium necrophorum*	aerosolized tobramycin
BS21 (II)	30	M	N1303K/M1V	68	23.8	*P. aeruginosa, S. epidermidis, P. denticola, Rothia dentocariosa, S. pseudopneumoniae*	aerosolized colistimethate, azithromycin
BS24 (II)	26	F	N1303K/3849+10kbC›T	50	19.8	*P. aeruginosa, S. epidermidis, V. parvula, S. peroris, S. sanguinis*	aerosolized colistimethate, azithromycin
BS30 (II)	25	M	F508del/UN	43	18.1	*P. aeruginosa, S. epidermidis, R. dentocariosa, A. odontolyticus, S.*	aerosolized colistimethate

Study ID	Age	Gender	CFTR Genotype	FEV_1	BMI	Microflora	Maintenance antimicrobial therapy
						pseudopneumoniae	
GS10 (II)	50	M	F508del/ F508del	57	19.2	*Staphylococcus* coagulase negative, *Streptococcus viridans*, *P. aeruginosa*, *A. odontolyticus*, *Bifidobacterium longum*, *G. haemolysans*, *A. fumigatus*	aerosolized colistimethate, aerosolized tobramycin, azithromycin
GS11 (II)	24	M	F508del/N1303K	42	24.4	*P. melaninogenica*, *Gemella sanguinis*, *S. parasanguinis*, *S. salivarius*, *P. denticola*	azithromycin
GS20 (II)	20	F	3849+10KbC>T/2118DEL4	67	19.7	*S. aureus* , *S. viridans*, *P. nanceiensis*, *P. melaninogenica*	aerosolized tobramycin, azithromycin
GS21 (II)	42	M	F508del/F508del	55	22.5	*S. aureus*, *S. viridans*, *Prevotella oris*, *P. nanceiensis*, *P. nigrescens*, *C. albicans*	none
MS3 (II)	25	F	F508del/G178R	56	21.4	*P. aeruginosa*, *S. aureus*, *Pseudomonas* spp, *Prevotella pallens*, *P. melaninogenica*, *S. parasanguinis*, *S. oralis*	aerosolized tobramycin,
MS4 (II)	42	M	F508del/3199del6/I148T	47	23.1	*S. aureus*, *P. aeruginosa*, *V. parvula*	aerosolized colistimethate
MS5 (II)	30	M	F508del/F508del	43	20.7	*P. aeruginosa*, *V. parvula*, *P. salivae*, *S. peroris*, *Streptococcus infantis*	aerosolized tobramycin, azithromycin
MS6 (II)	54	M	2789+5G >A/2789+5G >A	52	26.7	*P. aeruginosa*, *Abiotrophia defectiva*	azithromycin
MS7 (II)	52	M	2789+5G>A/1602delCT	55	24.3	*P. aeruginosa*, *A. odontolyticus*, *Lactobacillus gasseri*, *A. fumigatus*	azithromycin
MS14 (II)	11	F	F508del/G85Ee	44	15.4	*P. aeruginosa*, *S. aureus*	aerosolized tobramycin, azithromycin
BS3 (III)	26	F	F508del/F508del	29	22.0	*Burkholderia cepacia* complex, *S. aureus*	aerosolized colistimethate, aerosolized tobramycin, azithromycin
BS19 (III)	35	M	F508del/W1282X	37	24.9	*S. aureus*, *P. denticola*, *S. oralis*, *S. sanguinis*, *H.*	aerosolized colistimethate, azithromycin

Study ID	Age	Gender	CFTR Genotype	FEV_1	BMI	Microflora	Maintenance antimicrobial therapy
						parainfluenzae, Candida parapsilosis, C. albicans, A. fumigatus	
BS25 (III)	29	F	F508del/G551D	39	25.5	*B. cepacia* complex, *S. aureus, S. marcescens, R. mucilaginosa, G. sanguinis, G. bergeri, N. mucosa, H. parainfluenzae, H. influenzae, S. oralis*	none
BS39 (III)	21	M	F508del/F508del	28	20.5	*S. aureus, A. xylosoxidans, S. epidermidis, Haemophilus parahaemolyticus, S. sanguinis, Streptococcus constellatus, S. pseudopneumoniae, Neisseria subflava, R. dentocariosa, R. mucillaginosa, C. sputigena, G. haemolysans, C. albicans*	aerosolized colistimethate, aerosolized tobramycin, azithromycin
BS51 (III)	36	M	2789+5G>A/F508del	38	21.0	*A. xylosoxidans, P. aeruginosa, S. epidermidis, N. mucosa, A. odontolyticus, C. albicans*	aerosolized colistimethate, azithromycin
BS71 (III)	18	F	F508del/F508del	23	18.0	*P. aeruginosa, S. aureus, C. albicans*	aerosolized colistimethate, azithromycin
BS85 (III)	34	M	F508del/1259insA	21	18.8	*P. aeruginosa, Enterococcus faecalis, C. albicans, Aspergillus flavus, A. fumigatus*	aerosolized colistimethate, azithromycin
GS14 (III)	26	M	F508del/G85E	37	22.1	*S. aureus, A. xylosoxydans*	aerosolized colistimethate, azithromycin
MS10 (III)	29	M	F508del/F508del	25	24.3	*P. aeruginosa , S. maltophilia, V. parvula, S. pneumoniae, S. peroris*	aerosolized colistimethate, azithromycin

[a]In parenthesis, the FEV_1 group: I = normal/mild ($FEV_1\% > 70$); II = moderate ($70 \geq FEV_1\% \geq 40$); III = severe ($FEV_1\% < 40$).
