# Supplementary material for: Changes in Cystic Fibrosis Airway Microbial Community Associated with a Severe Decline in Lung Function
Source: PLoS One. 2015 Apr 21;10(4):e0124348. doi: 10.1371/journal.pone.0124348 (PMC4405530; doi:10.1371/journal.pone.0124348)
Supplement: S2 Table — (PDF) [file pone.0124348.s004.pdf]

Table S2. Characteristics of the substantial decliners' cohort at the enrollment in the study.

Study ID[a]	Age (years)	Gender	CFTR Genotype	FEV_1 (%predicted)	BMI	Microflora	Maintenance antimicrobial therapy
BSD9 (I)	23	F	F508del/1717-1G›A	84	21.0	*Mycobacterium abscessus, Veillonella atypica, Prevotella salivae, Pseudallescheria boydii*	aerosolized colistimethate, aerosolized tobramycin
BSD10 (I)	23	F	F508del/1717-1G›A	84	21.0	*M. abscessus, Prevotella melaninogenica, P. boydii*	aerosolized colistimethate aerosolized tobramycin
BSD13 (I)	29	M	F508del/G542X	71	22.0	*Pseudomonas aeruginosa, Veillonella dispar, Candida albicans*	aerosolized tobramycin
BSD22 (I)	21	F	F508del/G85E	81	22.1	*Staphylococcus aureus, Rothia mucilaginosa, Prevotella denticola, Neisseria perflava, Neisseria macacae, Neisseria mucosa, Haemophilus parainfluenzae, Streptococcus salivarius, Candida glabrata*	aerosolized tobramycin, azithromycin
BSD28 (I)	20	M	F508del/F508del	75	23.0	*S. aureus, Streptococcus viridans, Bacillus licheniformis, Neosartoria pseudofischeri*	aerosolized colistimethate, aerosolized tobramycin, azithromycin
GSD5 (I)	24	M	F508del/12491G>A	72	22.7	*Pseudomonas* spp., *S. aureus, S. viridians, Prevotella intermedia, Streptococcus pneumoniae*	none
GSD6 (I)	17	F	F508del/F508del	91	20.2	*S. aureus, Prevotella nanceiensis, Streptococcus peroris, Streptococcus pseudopneumoniae, S. pneumoniae, Aspergillus fumigatus*	none
GSD7 (I)	37	F	G542X/D1152H	78	19.4	*S. viridans, Stenotrophomonas maltophilia, P. nanceiensis, S. pseudopneumoniae, Streptococcus oralis, Granulicatella elegans, Prevotella nigrescens*	none

Study ID	Age	Gender	CFTR Genotype	FEV_1	BMI	Microflora	Maintenance antimicrobial therapy
						Actinomyces graevenitzii, *S. pneumoniae*	
GSD8 (I)	13	F	F508del/E585X	74	18.2	*S. maltophilia*, *P. aeruginosa*, *S. viridans*, *S. aureus*, *P. nanceiensis*, *G. sanguinis*	none
GSD9 (I)	10	F	2183AA>G/I148-3199DEL6	77	17.9	*P. aeruginosa*, *S. aureus*, *Actinomyces odontolyticus*	aerosolized colistimethate, azithromycin
GSD17 (I)	18	M	F508del/F508del	85	20.7	*S. aureus*, *Ralstonia* spp., *Delftia acidovorans*, *P. nanceiensis*, *Capnocytophaga ochracea*, *C. albicans*	none
MSD2 (I)	28	F	N1303K/L1065P	75	21.5	*P. aeruginosa*, *S. aureus*, *Lactobacillus suebicus*, *P. nigrescens*, *S. oralis*, *P. pseudopneumoniae*. *A. fumigatus*	aerosolized tobramycin, azithromycin
MSD8 (I)	16	M	F508del/N1303K	75	19.8	*S. aureus*, *A. fumigatus*	aerosolized aztreonam
BSD11 (II)	24	F	G542X/UN	60	21.6	*P. aeruginosa*, *S. aureus*, *P. melaninogenica*, *S. salivarius*	aerosolized colistimethate, aerosolized tobramycin, azithromycin
BSD12 (II)	17	F	G542X/W1282R	49	17.9	*P. aeruginosa*, *R. mucilaginosa*, *P. denticola*, *S. oralis*. *C. albicans*	aerosolized tobramycin, azithromycin
BSD14 (II)	18	F	F508del/R533X	69	20.0	*P. aeruginosa*, *S. aureus*, *R. mucilaginosa*, *P. denticola*, *V. atypica*, *P. melaninogenica*, *Neisseria flava*, *N. macacae*, *C. albicans*	aerosolized colistimethate
BSD16 (II)	28	F	F508del/4016insT	53	19.1	*P. aeruginosa*, *R. mucilaginosa*, *Actinomyces* spp., *S. peroris*, *C. albicans*	aerosolized aztreonam, aerosolized colistimethate
BSD18 (II)	18	F	G542X/N1303K	54	20.8	*S. aureus*, *R. mucilaginosa*, *Rothia aeria*, *H. parainfluenzae*, *S. oralis*, *S. pseudopneumoniae*	aerosolized tobramycin
BSD23 (II)	16	M	F508del/G542X	53	16.0	*P. aeruginosa*	aerosolized tobramycin

Study ID	Age	Gender	CFTR Genotype	FEV$_1$	BMI	Microflora	Maintenance antimicrobial therapy
						S. pseudopneumoniae, C. albicans	
GSD12 (II)	59	F	F508del/F508del	44	21.9	*S. viridans, S. aureus,* Enterobacteriacae, *P. aeruginosa*	azithromycin
GSD13 (II)	20	F	F508del/F508del	67	20.7	*S. aureus, P. melaninogenica, S. pneumoniae, C. albicans*	azithromycin
GSD22 (II)	30	M	DEL2,3(21KB)/L1077P	46	18.5	*P. aeruginosa, Staphylococcus* coagulase negative, *P. melaninogenica*	aerosolized tobramycin, azithromycin
GSD23 (II)	25	F	F508del/F508del	42	17.4	*P. aeruginosa, Escherichia coli*	azithromycin
GSD24 (II)	48	F	F508del/F508del	35	17.3	*S. aureus, P. aeruginosa, S. viridans, P. denticola, S. pneumoniae*	aerosolized colistimethate, azithromycin
GSD25 (II)	32	M	F508del/G542X	37	21.5	*S. aureus, P. aeruginosa, S. viridans, Prevotella pallens, A. odontolyticus, Atopobium rimae*	aerosolized colistimethate, azithromycin
MSD9 (II)	12	F	F508del/F508del	53	19.3	*S. aureus, S. maltophilia, Granulicatella adiacens*	aerosolized aztreonam, aerosolized tobramycin
BSD15 (III)	46	F	F508del/UN	37	19.7	*P. aeruginosa, S. aureus, C. albicans, C. dubliniensis*	aerosolized colistimethate, azithromycin
BSD20 (III)	24	M	F508del/F508del	36	23.3	*P. aeruginosa, Enterococcus faecalis, S. maltophilia, Staphylococcus epidermidis, R. mucilaginosa, P. salivae, Veillonella parvula, S. pseudopneumoniae, C. parapsilosis, C. albicans, A. fumigatus*	aerosolized aztreonam, aerosolized colistimethate
BSD37 (III)	24	F	F508del/UN	39	20.5	*Achromobacter xylosoxidans, Corynebacterium amicolatum, R. mucilaginosa, Rothia dentocariosa, V. parvula, P. denticola, S. salivarius, S. pseudopneumoniae, Scedosporium prolificans*	aerosolized colistimethate, aerosolized tobramycin

Study ID	Age	Gender	CFTR Genotype	FEV_1	BMI	Microflora	Maintenance antimicrobial therapy
BSD38 (III)	18	M	N1303K/F508del	35	16.6	*A. xylosoxidans*, *P. aeruginosa*, *V. dispar*, *Bifidobacterium breve* *R. mucillaginosa*, *Streptococcus gordonii*, *S. pseudopneumoniae*, *S. peroris*, *Streptococcus constellatus*	aerosolized tobramycin
BSD49 (III)	26	M	N1303K/G85E	29	19.9	*Burkolderia cepacia* complex, *P. aeruginosa*, *P. denticola*, *P. melaninogenica*, *Bifidobacterium dentum*, *Lactobacillus gasseri*, *Peptoniphilus haeri*	aerosolized colistimethate
GSD15 (III)	29	M	F508del/F508del	36	27.1	*S. aureus*, *P. aeruginosa*, *A. xylosoxydans*, *Cryseobacterium* spp.	aerosolized colistimethate, azithromycin
GSD26 (III)	45	M	F508del/H939R+H949L	28	19.2	*P. aeruginosa*, *P. melaninogenica*, *Parvimonas micra*, *Campylobacter concisus*, *A. odontolyticus*	aerosolized colistimethate
GSD27 (III)	25	M	F508del/F508del	29	22.5	*S. aureus*, *P. aeruginosa*, *A. xylosoxidans*, *Streptococcus spp.*, *S. oralis*, *S. salivarius*, *B. breve*	none
MSD11 (III)	31	M	4169delCTAAGCC/E585X	33	11.4	*A. xylosoxidans*, *S. pneumoniae*, *S. peroris*	aerosolized tobramycin, azithromycin
MSD12 (III)	37	F	F508del/del Exon 2	32	25.9	*P. aeruginosa*, *S. pneumoniae*	aerosolized colistimethate, azithromycin
MSD13 (III)	18	M	1898+1G>A/1898+1G>A	33	16.3	*P. aeruginosa*, *S. aureus*	azithromycin
MSD15 (III)	49	M	G1244E/del Exons 14a, 14b,15, 16, 17a, 17b	32	15.4	*P. aeruginosa*, *S. aureus*, *C. albicans*	azithromycin

[a]In parenthesis, the FEV_1 group: I = normal/mild ($FEV_1\% > 70$); II = moderate ($70 \geq FEV_1\% \geq 40$); III = severe ($FEV_1\% < 40$)
